# Surgical Operations in Private Practice

**Published:** 1905-04-01

**Authors:** John Aikman


### Hospital Clinics.

### SURGICAL OPERATIONS IN PRIVATE PRACTICE.

By John Aikman, M.D., C.M., Glas.

### VI.—Deformities.

While the treatment of some deformities is among the more advanced triumphs of modern surgery, and reaches its highest perfection at the hands of specialists, there are some operations which, for reasons of economy, should be undertaken by the general practitioner. The treatment of the simpler forms of hare-lip does not involve more manual dexterity than he ought to possess; the daylight operation for club-foot brings it under the same category. In each case there are duties in the way of preparation and selection which are worthy of consideration. Preparation has more reference to hare-lip, though the same methods apply to all deformities to which children are liable in the earlier months of their life. Selection is more difficult in cases of club-foot, for infantile paralyses play their part and experience steps in to make the choice and extent of interference a large factor in treatment. When tendons are divided the muscles waste beyond hope of repair; a severed tendo Achillis may cure a club-foot which the patient cannot remember, but it leaves him without a calf, which is a very present memory. The least satisfactory results attend operations in which the deformity appears some time after birth, and is the result of anterior poliomyelitis. It is a safe rule to overdo the operation for hare-lip; and to underdo the operation for club-foot.

Children born with a hare-lip are deprived of the nourishment from the mother's breast, and all artificial feeding is bereft of its due of salivary digestion. Milk and water requires little saliva, but it should be churned into minute particles by the action of the mouth. Dilution is one means of atonement, and it should not be by means of barley water nor any farinaceous material.

Correct preparation by means of diet is so important to the success of the operation that I append a few remarks. The following scale is reasonable for children whose digestion is naturally impaired:—

| | | | | | | | | | | | |
|---|---|---|---|---|---|---|---|---|---|---|---|
| Months ... ... | 0 | 1 | 2 | 3 | 4 | 5 | 6 | 7 | 8 | 9 | |
| Water ... ... | 9 | 8 | 7 | 6 | 5 | 4 | 3 | 2 | 1 | 0 | parts. |
| Cow's milk ... | 3 | 4 | 5 | 6 | 7 | 8 | 9 | 10 | 11 | 12 | parts. |

For each meal: First week, 12 teaspoonfuls every two hours; third week, 16 teaspoonfuls every two hours; sixth week, 12 dessert spoonfuls every three hours; third month, 16 dessert spoonfuls every three hours; sixth month, 12 table spoonfuls every four hours; eighth month, 16 table spoonfuls every four hours.

In the earlier weeks water is the best diluent, but soon albumen water—the white of one egg beaten up in a pint of water—may be used. When the child is three months old, the gelatinous soup of chicken or veal, in proportions increasing from one table spoonful to three to a pint of water, keeps the milk in fine division and adds vigour to the child's power of repairing an injury. Some such preparation should be made by the general practitioner whether he is to be the operator or not.

It is well to wait for the advent of the first teeth before operating; the stage of irritation should be anticipated, but that is the only call for denying the patient the advantage of the fullest age. The skin should be disinfected by frequent washing with soap and water, for all antiseptic precautions will be frustrated by the discharges from the nose and mouth; nevertheless, primary union of the operation wound should occur.

The details of all operations must be gathered from practical experience aided by the text-books, but there are some accessories which are helpful. It is well, after the first 24 hours, to remove all the crusts which gather round the line of incision and stitches by means of solution of peroxide of hydrogen. Such a procedure does no harm, while it keeps the after-scar at a minimum. The lip may be freely wetted many times a day, and left wet till much effervescence has taken place; after which the crusts will come off easily. It does not affect the silkworm gut, which is the best form of suture. Crusts from the nasal secretion, the saliva, and food are easily removed by its agency; moreover, it is antiseptic. Stitches may be left in for eight days.

In operations for club-foot the open method is best. The tendon is exposed in a situation in which the scar is free from the pressure of the boot, and the damage done is less than by subcutaneous division if perfect asepsis is insured. Plaster of Paris, or water-glass bandages, secure the foot in its new position for a reasonable time, and if the division is well judged, no expensive mechanical apparatus is required.

These minor operations stand in contrast in the fact that free division, and the abolition of tension secure the best result in hare-lip; while in club-foot, the smaller division leaves the stronger foot and the more perfect muscular support, even if the first operation has to be supplemented by other active measures later on.

